# Suicide and death by other causes among patients with a severe mental illness: cohort study comparing risks among patients discharged from inpatient care *v*. those treated in the community

**DOI:** 10.1017/S2045796022000075

**Published:** 2022-05-06

**Authors:** R. Musgrove, M. J. Carr, N. Kapur, C. A. Chew-Graham, F. Mughal, D. M. Ashcroft, R. T. Webb

**Affiliations:** 1 NIHR Greater Manchester Patient Safety Translational Research Centre, Centre for Mental Health and Safety, Manchester Academic Health Science Centre, The University of Manchester, Manchester, UK; 2 NIHR Greater Manchester Patient Safety Translational Research Centre, Centre for Pharmacoepidemiology and Drug Safety, Manchester Academic Health Science Centre, The University of Manchester, Manchester, UK; 3 NIHR Greater Manchester Patient Safety Translational Research Centre, Centre for Mental Health and Safety, Manchester Academic Health Science Centre, The University of Manchester and Greater Manchester Mental Health NHS Foundation Trust, Manchester, UK; 4 School of Medicine, Keele University, Keele, UK; 5 School of Medicine, Keele University, Keele, UK; NIHR Greater Manchester Patient Safety Translational Research Centre, University of Manchester, Manchester, UK and Unit of Academic Primary Care, University of Warwick, Coventry, UK

**Keywords:** Epidemiology, suicide, inpatient psychiatry, community mental health, schizophrenia

## Abstract

**Aims:**

People diagnosed with a severe mental illness (SMI) are at elevated risk of dying prematurely compared to the general population. We aimed to understand the additional risk among people with SMI after discharge from inpatient psychiatric care, when many patients experience an acute phase of their illness.

**Methods:**

In the Clinical Practice Research Datalink (CPRD) GOLD and Aurum datasets, adults aged 18 years and older who were discharged from psychiatric inpatient care in England between 2001 and 2018 with primary diagnoses of SMI (schizophrenia, bipolar disorder, other psychoses) were matched by age and gender with up to five individuals with SMI and without recent hospital stays. Using survival analysis approaches, cumulative incidence and adjusted hazard ratios were estimated for all-cause mortality, external and natural causes of death, and suicide. All analyses were stratified by younger, middle and older ages and also by gender.

**Results:**

In the year after their discharge, the risk of dying by all causes examined was higher than among individuals with SMI who had not received inpatient psychiatric care recently. Suicide risk was 11.6 times (95% CI 6.4–20.9) higher in the first 3 months and remained greater at 2–5 years after discharge (HR 2.3, 1.7–3.2). This risk elevation remained after adjustment for self-harm in the 6 months prior to the discharge date. The relative risk of dying by natural causes was raised in the first 3 months (HR 1.6, 1.3–1.9), with no evidence of elevation during the second year following discharge.

**Conclusions:**

There is an additional risk of death by suicide and natural causes for people with SMI who have been recently discharged from inpatient care over and above the general risk among people with the same diagnosis who have not recently been treated as an inpatient. This mortality gap shows the importance of continued focus, following discharge, on individuals who require inpatient care.

## Introduction

People diagnosed with severe mental illnesses (SMIs), including schizophrenia, bipolar affective disorder or other psychoses (NHS England, 2018), have a life expectancy that is 10–20 years lower than the population average (Wahlbeck *et al*., 2011; Hayes *et al*., 2017). Suicide risk is particularly raised following recent discharge from inpatient psychiatric care (Chung *et al*., 2017, 2019). However, a UK study estimated that almost 80% of life years lost in people with SMI were from natural causes of death (Jayatilleke *et al*., 2017). This is thought to be due to social risk factors, poorer access to healthcare and inadequate adherence to, and iatrogenic effects of, medication (Thornicroft, 2011). The UK's National Health Service (NHS) primary and secondary care services have targets to offer physical health checks and extra support for people with SMI diagnoses aiming to reduce this gap (NHS England and British Medical Association, 2019).

Previous comparisons have mostly been made between people diagnosed with an SMI, usually identified by hospital records, and the general population (Crump *et al*., 2013). Less is understood about mortality risk linked with an acute hospitalised illness phase compared to community-based treatment. Although historically, long-term hospitalisation was common for people with SMIs, this has been largely been replaced with community-based care and short-term hospital care, if needed, during acute illness phases (Killaspy, 2007). Greater suicide risk elevation among patients discharged from inpatient psychiatric care than for those with only outpatient visits in the previous year, *v*. persons not receiving any psychiatric treatment, has been reported from Denmark (Hjorthøj *et al*., 2014) and Taiwan (Yeh *et al*., 2017). These studies highlighted differential suicide risk among mental healthcare patients according to patients' treatment settings. However, they were not specific to SMI and did not consider natural deaths. Furthermore, as case-control designs were implemented, variability in relative risks across post-discharge follow-up periods could not be examined.

The only previous study to have compared people with an SMI in different healthcare settings found no difference in standardised mortality ratio (SMR) estimates when they examined a primary care cohort of persons with and without history of inpatient psychiatric care, as indicated in linked secondary care records in Wales (John *et al*., 2018). The authors noted that their findings did not point to a difference in disease severity between the two study cohorts. This investigation did not, however, specifically consider the post-discharge period.

No longitudinal studies of people diagnosed with an SMI have previously directly compared mortality risk in the post-discharge period with other SMI-diagnosed patients without a recent inpatient stay. This study has augmented the published evidence-base by enhancing our understanding of the specific excess mortality risk among recently discharged SMI patients *v*. individuals with the same disorders who are treated in community settings. This will enable more appropriate targeting of resources and commissioning of services.

We aimed to compare risks of external causes of death (including suicide, accident, poisoning and assault) and natural causes, between persons recently discharged from inpatient psychiatric care with SMI to their peers of the same age and gender and with similar diagnoses and without recent hospitalisation. We estimated: (a) absolute risk of all-cause and cause-specific mortality in SMI patients with and without recent inpatient care at one year post-discharge; (b) relative risk between these two groups at 3 months, 1 year and later follow-up periods; and (c) relative risk adjusted for known risk factors: non-fatal self-harm (for suicide risk) and physical health comorbidity (for risk of dying by natural causes). Gender- and age-specific estimates were also calculated. We hypothesised that risks for dying by suicide and other external causes would be higher in the first year post-discharge, later dropping to similar levels as in the community cohort, and that early post-discharge risk elevation would be partly explained by recent healthcare presentation for non-fatal self-harm, which is the strongest known risk factor for suicide. We did not expect to identify any difference between the two groups in their respective risks of dying by natural causes.

## Method

### Data sources

We utilised interlinked primary and secondary care health records in the Clinical Practice Research Datalink (CPRD) in England. The CPRD is broadly representative of the national population, containing primary care records from 16 million patients registered with general practices that use Vision^®^ and EMIS Web^®^ software in its GOLD and Aurum datasets (Herrett *et al*., 2015; Wolf *et al*., 2019). The CPRD has ethical approval from the Health Research Authority to support research using anonymised patient data (CPRD, 2019). Electronic health records pertaining to inpatient admissions from NHS hospitals and NHS care received in privately funded healthcare facilities were linked using the Hospital Episode Statistics Admitted Patient Care (HES APC) dataset. Additional linkage was made to mortality records from the Office for National Statistics (ONS), and to the 2015 English Index of Multiple Deprivation (IMD) quintiles (Ministry of Housing, 2019). Further information on these datasets can be found in the Appendix (p2).

### Patient populations and study design

In this matched cohort study, exposed cohort members were adults with an inpatient stay under the care of a psychiatry consultant with a primary diagnosis of schizophrenia, bipolar disorder or other psychoses (International Classification of Diseases, 10th revision, codes F20–F31, F32.3, F33.3) discharged between 1 January 2001 and 31 May 2018. Patients were 18 years or older at discharge. The index date was set as the date of first discharge during the study's observation period.

Up to five comparator patients (per discharged patient) diagnosed with an SMI were identified in primary care records using relevant routinely entered clinical Read and SNOMED codes. Codes were agreed by clinical academics (CC-G and FM: GPs; NK: psychiatrist) using the SMI definition given in the NHS Quality Outcomes Framework (QOF) financial incentive scheme in primary care (NHS England and British Medical Association, 2019). Patients were matched on gender and birth year. Patients were excluded if they had experienced inpatient psychiatric care during the 3 years before the index date of their matched discharged patient. Follow-up started at index date for up to a year until earliest of: death, recorded out-of-practice transfer or the study's final observation date (31 May 2019). See Appendix Table S1, p3 for further details.

### Classification of outcomes and covariates

Cause of death was ascertained via ONS mortality records – external causes: ICD-10 codes V01–Y98; natural deaths: all other codes. Suicides, including unnatural deaths of undetermined intent, as is convention in the UK (Linsley *et al*., 2001), were classified as X60–X84, Y10–Y34 (excluding Y33.9), Y87.0 and Y87.2. Non-fatal self-harm episodes, defined as ‘any act of self-poisoning or self-injury carried out by a person, irrespective of their motivation’ (National Institute for Health Care and Excellence (NICE), 2011) were identified using primary care codes applied in a previous CPRD-based study (Carr *et al*., 2021) and ICD-10 codes X60–X84 denoting hospital admissions following intentional self-harm. Comorbidities at baseline were classified using code lists based on the Charlson Comorbidity Index (Charlson *et al*., 1987), initially adapted by Khan *et al*. (2010); see Appendix (p4) for list of conditions. Practice locality and residential neighbourhood IMD quintiles were also utilised (see Appendix p2 for further information).

### Statistical analyses

All analyses were performed using Stata software version 16 (StataCorp, 2019). Cumulative incidence curves and estimates for each outcome at 1-year post-discharge were generated for all cohort members and by age (18–39, 40–64 and 65 years and older) and gender. Cox regression (Cox, 1972) models were fitted to conduct survival analyses for each examined outcome over the first 5 years post-discharge. Hazard ratios were adjusted for residential neighbourhood and practice locality IMD quintiles and SMI diagnostic category (schizophrenia and related disorders, bipolar disorder, other affective psychoses). Hazard ratios were estimated separately for the following follow-up periods: under 3 months, 3 months to a year, during the second year and from the second anniversary to 5 years post-discharge. This approach was taken to account for greatly elevated suicide rates soon after discharge (Chung *et al*., 2017, 2019), because the Cox regression model assumes that associations remain constant throughout follow-up (Cox, 1972). Interaction terms for age group and gender were additionally fitted for the first year post-discharge and, if average hazard ratios varied significantly, stratified estimates were calculated. Finally, models for suicide were run with adjustments for prior healthcare presentation for self-harm and presence of comorbidities and for natural causes with adjustment for comorbidities.

This study is reported in line with RECORD guidance for reporting of observational studies conducted using routinely collected data (Appendix Table S2, p5) (Benchimol *et al*., 2015). An advisory group of mental health service users and carers supported by the NIHR Greater Manchester Patient Safety Research Centre (GM PSTRC) provided feedback on plans and contributed to the interpretation of the study's findings.

## Results

### Descriptive information

The discharged cohort consisted of 23 942 people, 60% with a primary diagnosis of schizophrenia and related disorders, 25% with bipolar disorder and 15% with other affective psychoses. The comparison cohort of 119 360 persons had a broadly similar diagnostic profile (Table 1). The median age of cohort members was 46 years (IQR 29). Sixty per cent of discharged patients aged under 40 were male; two-thirds of those over 65 were female (see Appendix Fig. S1, p9, for full age distribution). Around half of cohort members lived in the two most deprived quintiles. Almost 7% of discharged patients had documented recent self-harm, compared to less than 1% in the community cohort.

**Table 1.** Socio-demographic and clinical profiles of discharged and matched community cohorts

	Discharged cohort *n* = 23 942		Matched community cohort *n* = 119 360	
	*N*	%	*n*	%
Gender				
Male	11 796	49.3	58 776	49.2
Female	12 146	50.7	60 584	50.8
Age at discharge (years)				
18–24	2442	10.2	12 104	10.1
25–34	4237	17.7	21 149	17.7
35–44	4590	19.2	22 915	19.2
45–54	4074	17.0	20 353	17.1
55–64	3230	13.5	16 141	13.5
65–74	2715	11.3	13 562	11.4
75–84	1957	8.2	9752	8.2
85+	697	2.9	3384	2.8
Ethnicity				
White	19 338	80.8	94 924	79.5
Black, Black British, Caribbean or African	2067	8.6	6219	5.2
Asian or Asian British	1376	5.7	5701	4.8
Mixed or multiple ethnic groups	362	1.5	1560	1.3
Other ethnic group	495	2.1	1976	1.7
Unknown	304	1.3	8980	7.5
Neighbourhood deprivation quintile (IMD)				
1 (Least deprived)	3286	13.7	17 784	14.9
2	3631	15.2	19 634	16.4
3	4277	17.9	22 359	18.7
4	5561	23.2	26 916	22.6
5 (Most deprived)	7187	30.0	32 667	27.4
Documented self-harm in previous 6 months				
	1619	6.8	1041	0.9
Number of comorbid conditions at baseline^a^				
0	18 874	78.8	90 824	76.1
1	3331	13.9	17 995	15.1
2	1135	4.7	6858	5.7
3 or more	602	2.5	3683	3.1
Primary diagnosis^b^				
Schizophrenia and related disorders	14 411	60.2	72 618	60.8
Bipolar disorder	5871	24.5	32 976	27.6
Other mood disorder with psychosis	3660	15.3	13 766	11.5

a Based on the Charlson classification.

b For the discharged cohort this was the primary diagnosis associated with the hospitalisation, for the community cohort it was the first identified SMI code in their primary care records.

### Absolute risk in discharged patients with SMI

**Fig. 1.**
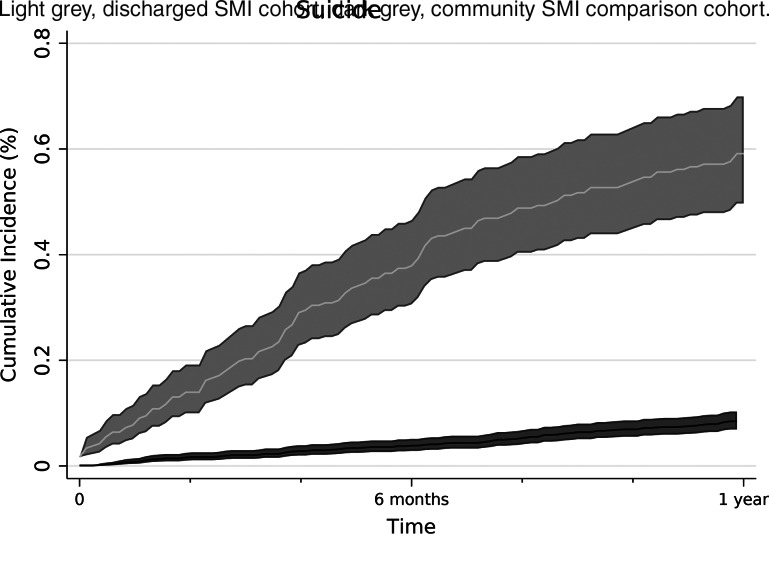
Cumulative incidence percentage values (and their 95% confidence intervals) of suicide in the first year post-discharge compared to individuals with a diagnosis of an SMI but without recent hospital admission.

The cumulative incidence of all-cause mortality in the first year post-discharge was 2.9% (95% CI 2.7–3.2) compared to 2.0% (1.9–2.1) in the community cohort. Natural causes comprised 73% of all deaths in the discharged cohort, absolute risk of 2.2% (2.0–2.4), compared to 1.8% (1.8–1.9) in the community group (Table 2, Appendix Figs S2 and S3, p9). The absolute risk of dying by suicide among discharged patients was 0.6% (0.5–0.7) compared to 0.1% (0.1–0.1) in the community group. The cumulative incidence of dying by an external cause, particularly suicide, increased steeply in the first 6 months post-discharge (Fig. 1, Appendix Fig. S4, p10). However, the suicide risk elevation immediately after discharge was not as pronounced as for the wider cohort of discharged people to which this SMI subset belongs (Appendix Fig. S5, p11). The risk of all-cause mortality overall was higher in women 2.4% (2.1–2.7) than in men (2.0%, 1.7–2.2). However, this was due to the difference in age distribution between the genders; when stratified by age, absolute risk was higher among men in each age stratum (Table 2). Suicide risk was the same in the younger and middle-aged adults (0.7%, 0.5–0.9) and 0.3% in older adults (0.2–0.4).

**Table 2.** Cumulative incidence percentage values for mortality outcomes at one year after discharge from inpatient psychiatric care by age group and gender

	All ages (18+)									Younger adults (18–39)								
	Total			Female			Male			Total			Female			Male		
	*n*	%* (95% CI)		*n*		%* (95% CI)	*n*	%* (95% CI)		*n*		%* (95% CI)	*n*		%* (95% CI)	*n*		%* (95% CI)
Discharged cohort	*N* = 23 942			*N* = 12 146			*N* = 11 796			*N* = 8979			*N* = 3498			*N* = 5481		
All-cause mortality	632	2.9	(2.7–3.2)	328	3.0	(2.7–3.3)	304	2.9	(2.6–3.2)	76	1.0	(0.8–1.2)	23	0.7	(0.5–1.1)	53	1.1	(0.8–1.4)
Natural causes	463	2.2	(2.0–2.4)	260	2.4	(2.1–2.7)	203	2.0	(1.7–2.2)	13	0.2	(0.1–0.3)	–	–	–	–	–	–
External causes	169	0.8	(0.7–0.9)	68	0.6	(0.5–0.8)	101	1.0	(0.8–1.2)	63	0.8	(0.6–1)	19	0.6	(0.4–0.9)	44	0.9	(0.7–1.2)
Suicide	128	0.6	(0.5–0.7)	44	0.4	(0.3–0.5)	84	0.8	(0.6–1.0)	55	0.7	(0.5–0.9)	16	0.5	(0.3–0.8)	39	0.8	(0.6–1.1)
Matched comparison cohort	*N* = 119 360			*N* = 60 584			*N* = 58 776			*N* = 44 733			*N* = 17 428			*N* = 27 305		
All-cause mortality	2291	2.0	(1.9–2.1)	1351	2.3	(2.2–2.4)	940	1.7	(1.6–1.8)	125	0.3	(0.2–0.4)	32	0.2	(0.1–0.3)	93	0.4	(0.3–0.4)
Natural causes	2104	1.8	(1.8–1.9)	1282	2.2	(2.1–2.3)	822	1.5	(1.4–1.6)	44	0.1	(0.1–0.1)	12	0.1	(0–0.1)	32	0.1	(0.1–0.2)
External causes	187	0.2	(0.1–0.2)	69	0.1	(0.1–0.2)	118	0.2	(0.2–0.3)	81	0.2	(0.2–0.2)	20	0.1	(0.1–0.2)	61	0.2	(0.2–0.3)
Suicide	97	0.1	(0.1–0.1)	25	0.04	(0.03–0.06)	72	0.1	(0.1–0.2)	53	0.1	(0.1–0.2)	10	0.1	(0–0.1)	43	0.2	(0.1–0.2)

	Middle-aged adults (40–64)									Older adults (65+)								
	Total			Female			Male			Total			Female			Male		
	*n*	%* (95% CI)		*n*	%* (95% CI)		*n*	%* (95% CI)		*n*	%* (95% CI)		*n*	%* (95% CI)		*n*	%* (95% CI)	
Discharged cohort	*N* = 9594			*N* = 5040			*N* = 4554			*N* = 5369			*N* = 3608			*N* = 1761		
All-cause mortality	186	2.1	(1.8–2.4)	75	1.6	(1.3–2)	111	2.7	(2.2–3.2)	370	7.8	(7.1–8.6)	230	7.2	(6.3–8.1)	140	9.0	(7.6–10.5)
Natural causes	107	1.2	(1–1.5)	47	1.0	(0.7–1.3)	60	1.5	(1.1–1.9)	343	7.2	(6.5–8.0)	209	6.5	(5.7–7.4)	134	8.6	(7.3–10.1)
External causes	79	0.9	(0.7–1.1)	28	0.6	(0.4–0.8)	51	1.2	(0.9–1.6)	27	0.6	(0.4–0.8)	21	0.7	(0.4–1)	6	0.4	(0.2–0.8)
Suicide	60	0.7	(0.5–0.9)	19	0.4	(0.3–0.6)	41	1.0	(0.7–1.3)	13	0.3	(0.2–0.4)	–	–	–	–	–	–
Matched comparison cohort	*N* = 47 929			*N* = 25 180			*N* = 22 749			*N* = 26 698			*N* = 17 976			*N* = 8722		
All–cause mortality	453	1.0	(0.9–1.1)	197	0.8	(0.7–0.9)	256	1.2	(1–1.3)	1713	6.6	(6.3–6.9)	1122	6.4	(6–6.8)	591	6.9	(6.4–7.5)
Natural causes	402	0.9	(0.8–1)	182	0.7	(0.6–0.9)	220	1.0	(0.9–1.1)	1658	6.4	(6.1–6.7)	1088	6.2	(5.9–6.6)	570	6.7	(6.2–7.2)
External causes	51	0.1	(0.1–0.1)	15	0.1	(0–0.1)	36	0.2	(0.1–0.2)	55	0.2	(0.2–0.3)	34	0.2	(0.1–0.3)	21	0.2	(0.2–0.4)
Suicide	26	0.1	(0–0.1)	6	0.02	(0–0.1)	20	0.1	(0.1–0.1)	18	0.1	(0–0.1)	–	–	–	–	–	–

### Relative risk by time elapsed since discharge

**Fig. 2.**
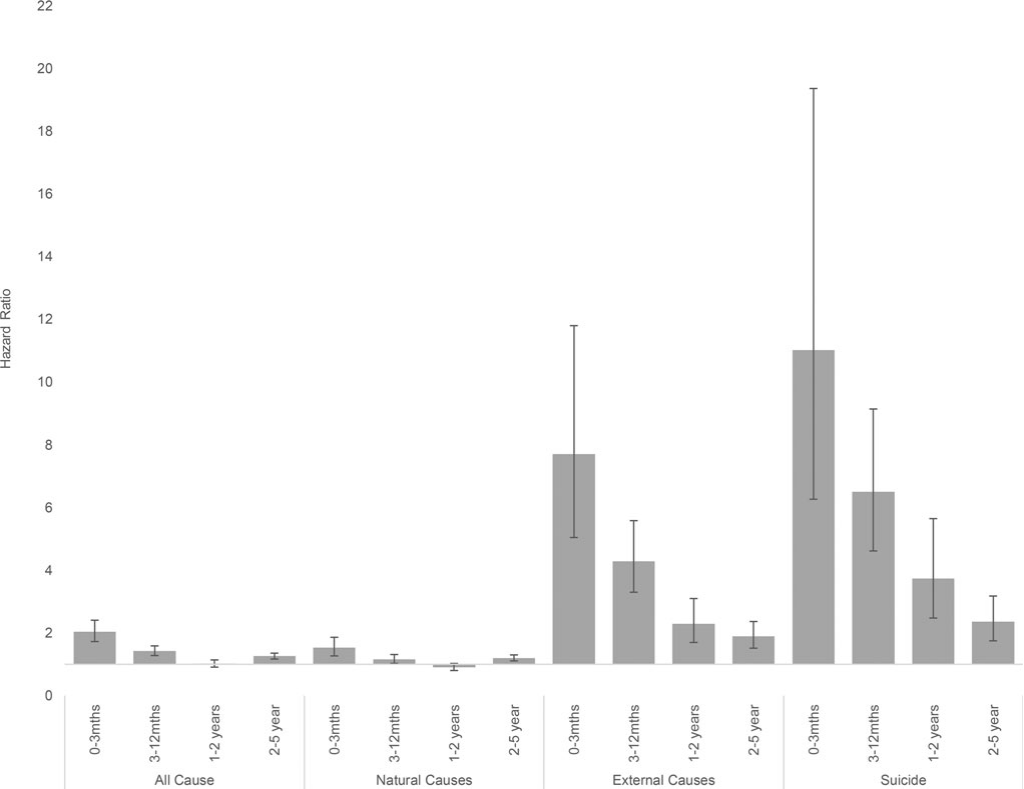
Hazard ratios by cause of death and post-discharge follow-up time period (adjusted for deprivation quintile and SMI subgroup).

Relative risk of death by each cause of death category was higher in the discharged cohort *v*. the community cohort at 3 months and 1 year post-discharge (Fig. 2, Appendix Table S3, p12). The relative risk was highest for suicide; 11.6 times the level in the community SMI cohort (95% CI 6.4–20.9) during the first 3 months post-discharge. The relative risk of suicide attenuated over time but remained over twice as high beyond the first 2 years post-discharge (HR 2.3, 1.7–3.2). Death by natural causes was also elevated in the first 3 months (HR 1.6, 1.3–1.9) compared to the rest of the first year (HR 1.2, 1.0–1.3) post-discharge. During the second follow-up year, there was no evidence of difference between the two cohorts, although there was evidence of increased risk at 2–5 years (HR 1.2, 1.1–1.3).

### Gender- and age-specific relative risks

**Fig. 3.**
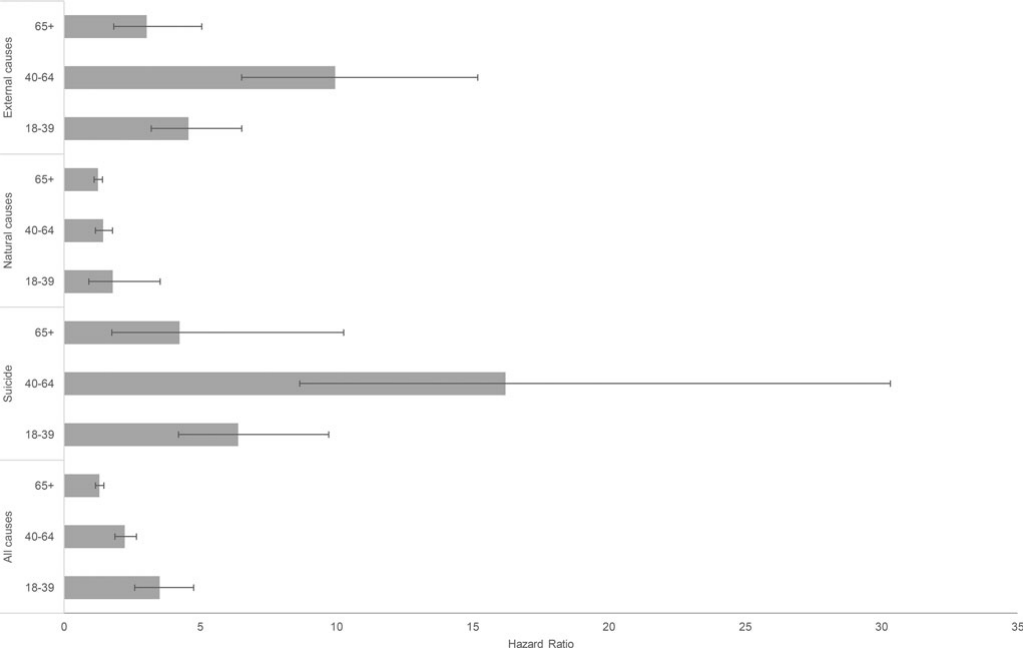
Hazard ratios by cause of death and age group at 1 year post-discharge (adjusted for deprivation quintile and SMI subgroup).

Average relative risks stratified by age and specific to cause of death in the first-year post-discharge are presented in Fig. 3. Tests for interaction by age group indicated that younger adults had a higher relative risk for all-cause mortality than middle-aged adults (interaction test: *p* = 0.01). Older adults had a lower relative risk than middle-aged adults, *p* < 0.001. These differences were largely driven by the differential composition of external and natural causes by age. Compared to middle-aged adults, younger adults had half the relative risk of dying by external causes (*p* = 0.01) and older adults under a third (*p* < 0.001). Relative risks for dying by natural causes did not vary significantly by age. Men had a higher relative risk for all-cause mortality at 1 year post-discharge (HR 1.9, 1.6–2.1) than women (HR 1.5, 1.3–1.6), *p* = 0.01. There was no evidence of variation in relative risk by gender for natural or external causes of death or for suicide.

### Multivariable adjustment

In the models examining suicide risk, additional adjustment for comorbidity and self-harm in the preceding 6 months attenuated the hazard ratio slightly from 11.6 (6.4–20.9) to 10.4 (5.7–19.0) during the first 3 months post-discharge. Table 3 provides details of HRs of each model including adjustment for deprivation quintiles which increased the risk somewhat. With adjustment for comorbidities at baseline the hazard ratio for natural causes increased marginally in the first 3 months from 1.6 (1.3–1.9) to 1.7 (1.4–2.1), with no material change after adjustment for deprivation. No change was observed in the remainder of the first post-discharge follow-up year.

**Table 3.** Cox models for suicide and natural causes of death adjusted for key risk factors

Variables adjusted for	Time after discharge (months)	Hazard ratio	95% CI
Death by suicide			
Unadjusted	0–3	9.7	(5.8–16)
	3–12	6.2	(4.5–8.6)
Deprivation (IMD practice and patient)	0–3	11.0	(6.3–19.4)
	3–12	6.5	(4.6–9.2)
Deprivation + SMI subgroup	0–3	11.6	(6.4–20.9)
	3–12	6.5	(4.6–9.2)
Deprivation + SMI subgroup + self–harm	0–3	10.4	(5.7–19)
	3–12	6.0	(4.2–8.5)
Deprivation + SMI subgroup + self–harm + comorbidities	0–3	10.4	(5.7–19)
	3–12	5.9	(4.1–8.4)
Natural causes			
Unadjusted	0–3	1.5	(1.3–1.9)
	3–12	1.2	(1–1.3)
Deprivation (IMD practice and patient)	0–3	1.5	(1.3–1.9)
	3–12	1.2	(1–1.3)
Deprivation + SMI subgroup	0–3	1.6	(1.3–1.9)
	3–12	1.2	(1–1.3)
Deprivation + SMI subgroup comorbidities at baseline	0–3	1.7	(1.4–2.1)
	3–12	1.2	(1.1–1.4)

## Discussion

During the year after discharge from inpatient psychiatric care, patients diagnosed with SMIs had a higher risk of dying by both external and natural causes than their SMI-diagnosed community-based counterparts. Over the first 3 months post-discharge, suicide risk was over 11 times higher *v*. individuals without recent hospital stays. However, suicide deaths were distributed throughout the 3 months rather than there being a large elevation in suicide risk in the first days and weeks post-discharge as reported previously (Chung *et al*., 2019; Bojanic *et al*., 2020). Suicide risk did not, as hypothesised, return to similar levels to the community SMI cohort after 1 year of follow-up, instead remaining at least double the risk at 2–5 years post-discharge. The enduring risk elevation was not explained by relative deprivation or recent self-harm episodes. The gap in risk of dying by external causes between the two study cohorts was greatest in middle-aged people and did not vary significantly by gender. The raised relative risk of death by natural causes was small in the first year post-discharge; nonetheless, the relative risk was most elevated in the first 3 months. No discernible difference in risk of dying by natural causes was found 1–2 years after discharge, although it was elevated slightly over the longer term.

### Interpretation

Observing elevated suicide risk within 3 months of discharge was expected based on previous meta-analyses (Chung *et al*., 2017, 2019). However, the less pronounced elevation in the first weeks may reflect the former policy of early follow-up for discharged patients subject to the Care Programme Approach, which prioritised patients with SMIs *v*. those discharged with other disorders (Schneider *et al*., 1999; NHS England and NHS Improvement, 2021). Nonetheless, suicide risk post-discharge was considerably higher than in patients treated in the community, indicating that current guidance on transition is not being fully implemented or is not wholly effective (NICE, 2016). Discharged patients are more likely to be in an acute phase of their illness and the experience of being admitted (involuntarily in some instances), adjusting to this new status and environment, disruption to social relationships and perceived stigma post-discharge may all contribute to heightened risk in some of these individuals (Owen-Smith *et al*., 2014).

We expected recent self-harm to attenuate the relative risk of suicide. However, due to its relatively low prevalence, 6.8% (discharged patients) *v*. 0.9% (comparison cohort), its confounding influence was weak. Furthermore, self-harm may be a less strong predictor of suicide risk among SMI patients *v*. its influence in the general population. Risks of suicide, and of dying prematurely, tend to be higher in relatively deprived areas (Cairns *et al*., 2017). However, patients from deprived quintiles were overrepresented in both SMI cohorts, and adjustment actually led to a slight increase in the relative risk of suicide, indicating a possible higher risk among less deprived discharged SMI patients. This concurs with findings reported from studies conducted in Denmark (Agerbo *et al*., 2001) and in England (Musgrove *et al*., 2021).

That suicide risk remained at least twice as high several years after discharge likely indicates greater underlying illness severity. Discharged patients may include more people who are inadequately supported to manage their condition, leaving them more vulnerable to suicide (Harris *et al*., 2019; Mutschler *et al*., 2019). Middle-aged people had the highest relative risk of dying by external causes in the first year, perhaps due to family and financial pressures (NCISH, 2021), which may be exacerbated by an inpatient admission. Early Intervention in Psychosis (EIP) services in the UK have traditionally targeted younger people, which may have left a gap in support for those who are middle-aged (Mitford *et al*., 2010; Greenfield *et al*., 2018).

The risk of dying by natural causes returned to similar levels after a year post-discharge, indicating that elevated risk may relate to circumstances pertaining to the hospital stay and transition rather than differences in the underlying risk of death from physical illness. It is conceivable that a loss of autonomy during hospitalisation and lack of appropriate assessment for physical health at discharge may lead to difficulties in managing health conditions. These issues have been identified in research among older adults discharged from general hospital inpatient care (Hestevik *et al*., 2019). Similar research examining the experiences of patients after psychiatric discharge would be informative.

### Strengths and limitations

This novel investigation directly compared risk among discharged patients with an SMI diagnosis *v*. individuals with these diagnoses identified in primary care records and without recent hospital stays. The CPRD enabled us to delineate a large, broadly nationally representative, study cohort with linkage between primary care and inpatient records.

The study, however, had some limitations. First, it relied on individuals being registered with the same GP for at least 6 months at index date. Although almost all people living in England are registered with a GP, those who are homeless or transient, demographic subgroups at greatest risk of dying prematurely, are less likely to have records in the study's dataset (John *et al*., 2018). The absolute risk values may therefore be underestimates. Second, one in seven of all discharged patients had an ‘unspecified’ diagnosis, meaning that our SMI cohort may have been incomplete. Post-hoc analysis estimated a higher suicide risk in this unspecified group than those with SMI, so we may have underestimated actual risks somewhat. Third, although higher suicide risk among involuntarily admitted patients has been reported (Kallert *et al*., 2008), such information was unavailable, which precluded examination of this subgroup. Furthermore, although almost all UK patients receive publicly-funded healthcare it is plausible that inclusion of privately funded care, if available, would have affected the findings somewhat. Fourth although comparison cohort members had an SMI diagnosis and were matched on age and gender, we could not assess the degree of severity within diagnoses or the current treatment type. Utilisation of validated, standardised severity measures for specific diagnoses requires information that is unavailable in routinely collected health records (Zimmerman *et al*., 2018). Finally, as some patients in the comparison cohort will have had historical inpatient stays, there is likely to be some exposure misclassification, which would attenuate the relative risk estimates towards unity (Copeland *et al*., 1977). Nevertheless, this is a useful comparison as it contains a broad range of individuals all of whom will be covered by NHS policies for people diagnosed with SMIs.

### Generalisability

As elevated suicide risk post-discharge is an international phenomenon, our findings are broadly generalisable. Discharge planning and follow-up feature in the policies of many nations (e.g. in the USA, Veterans Health Administration (2013); in Australia, NOUS Group (2018); in India, Gowda *et al*. (2019); and in Germany, Weiß *et al*. (2020)). However, comparisons between discharged and community-treated patient populations will be influenced considerably by the implementation of such policies. Thus, evaluative research of these specific interventions is needed. Where no transitional services exist we would expect a greater mortality gap between the two groups of SMI patients, and the development of such services is therefore a priority.

### Implications

Whilst the policy focus on patients discharged with SMIs in England may have reduced somewhat the marked elevation in suicide risk in the immediate post-discharge period for some individuals, the same support should be provided for all discharged patients (irrespective of their diagnosis). Additional support should be given to patients aged 39–64 who are at greatest risk of dying by external causes *v*. their community-treated counterparts with SMIs. From 2016, EIP services were expanded to support patients aged over 35 years (Clay *et al*., 2018). Further research investigating the impact of widening the eligible age range for this service on risk of dying by external causes is needed. Despite this policy emphasis, there remains an enduring elevation in mortality risk between people recently discharged from inpatient care and other patients diagnosed with SMIs who have not been recently hospitalised. The gap in risk estimates between these two diagnostically similar cohorts demonstrates the importance of continued focus on individuals for whom inpatient care may be necessary. Although admitted patients may be more acutely ill, we might expect a hospital stay to reduce onward suicide risk to a similar level to that for patients with the same diagnoses treated in community settings. Our findings indicate that this is not so. New approaches are needed to provide therapeutic care in the most appropriate setting and to support the transition back to the community if inpatient care is needed. The 2019 NHS Long Term Plan provides alternatives to admission including intensive home treatment support and crisis houses. It also includes the development of integrated primary care and community mental health teams to provide coordinated support to people with SMI (NHS England and NHS Improvement, 2019). Primary care has an important role in managing the day-to-day physical and mental health needs of these patients and effective coordination between providers is essential to ensure people receive appropriate support. Further qualitative research into patients' experiences of this challenging and risky transition would contribute to the commissioning of apposite, co-produced services aimed at reducing post-discharge risks.

## Data Availability

The clinical codes that were applied are available online at https://clinicalcodes.rss.mhs.man.ac.uk/. Data can only be accessed via application to the CPRD.

## Supplementary material

For supplementary material accompanying this paper visit https://doi.org/10.1017/S2045796022000075. click here to view supplementary material
