# Supplementary material for: Suicide and death by other causes among patients with a severe mental illness: cohort study comparing risks among patients discharged from inpatient care v. those treated in the community
Source: Epidemiol Psychiatr Sci. 2022 May 6;31:e32. doi: 10.1017/S2045796022000075 (PMC7612694; doi:10.1017/S2045796022000075)
Supplement: Supplementary file 1 [file S2045796022000075sup001.docx]

**Supplementary Appendix**

**Contents**

**Page 2** Datasets used with key links and references

**Page 3 Table S1** Delineation of the cohort of recently discharged patients

**Page 4** List of diagnostic categories included in the Charlson Comorbidity Index.

**Page 5 Table S2** RECORD Statement for observational studies

**Page 9** **Figure S1** Age distribution of SMI subgroup

**Page 9** **Figure S2, S3, S4** Cumulative incidence by cause of death in the first year post-discharge compared

to individuals with a diagnosis of an SMI but without recent hospital admission.

**Page 11 Figure S5** Graph showing the cumulative incidence of suicide in the first year after discharge

among all people discharged, irrespective of diagnosis.

**Page 12 Table S3** Unadjusted and adjusted hazard ratios by cause of death and follow-up time

**S1. Datasets used with key links and references**

**CPRD primary care dataset (GOLD)**

Herrett E, Gallagher AM, Bhaskaran K, et al. Data Resource Profile: Clinical Practice Research Datalink (CPRD). *Int J Epidemiol* 2015; **44**(3): 827-36. <https://academic.oup.com/ije/article/44/3/827/632531>

**CPRD primary care dataset (Aurum)**

Wolf A, Dedman D, Campbell J, et al. Data resource profile: Clinical Practice Research Datalink (CPRD) Aurum. *International Journal of Epidemiology* 2019; **dyz034**. <https://academic.oup.com/ije/article/48/6/1740/5374844>

**HES Secondary Care dataset**

The Admitted Patient Care or Inpatient dataset is part of a collection of datasets in the Hospital Episode Statistics (HES) data warehouse containing information on all admissions to English NHS hospitals. It is derived from the Commissioning Dataset (CDS) which is used as the basis of allocating payment for care provided by hospitals. It is administered by NHS Digital who facilitate the data-linkage to the CPRD.

NHS Digital. Hospital Episode Statistics - Hospital Admitted Patient Care Activity [Datafiles]. 2009-2019. <https://digital.nhs.uk/data-and-information/publications/statistical/hospital-admitted-patient-care-activity> (accessed Nov 14 2019).

Further information about the linkage process and quality checks can be found in the article below.

Padmanabhan S, Carty L, Cameron E, Ghosh RE, Williams R, Strongman H. Approach to record linkage of primary care data from Clinical Practice Research Datalink to other health-related patient data: overview and implications. *European Journal of Epidemiology* 2019; **34**(1): 91-9.

**Index of Multiple Deprivation (IMD)**

This is an area-level composite measure of relative deprivation encompassing these seven domains: health, income, employment, education, crime, barriers to housing and services, and living environment. It is available for patients’ residential neighbourhoods and practices’ locations, measured at the lower layer super output area (LSOA) level, with LSOAs being local geographical areas containing 1,000-3,000 residents. Where a residential neighbourhood IMD quintile was missing, (less than 0.1% of all cohort members), the practice’s IMD quintile was applied instead.

Ministry of Housing, Communities & Local Government,. English indices of deprivation [Intenet]. 26/09/2019 2019. <https://www.gov.uk/government/collections/english-indices-of-deprivation> (accessed Nov 25 2019).

**Office for National Statistics – Mortality Records**

<https://www.ons.gov.uk/peoplepopulationandcommunity/birthsdeathsandmarriages/deaths>

**Table S1- Delineation of the cohort of recently discharged patients with a primary diagnosis of a severe mental illness (SMI)**

A combined study dataset using the two CPRD datasets (Aurum and GOLD) was utilised, with a ‘bridging’ file applied to preclude double-counting of practices that had changed software provider. Patients in both cohorts were eligible for inclusion if their record was deemed acceptable for research and they were registered at a CPRD general practice for at least 6 months (183 days) before and including the index discharge date. Because of these strict eligibility criteria, up to 5 matched comparator patients were selected, which gave sufficient power for the primary analysis. Of those discharged, 99% had 5 eligible matches. 27 individuals without any eligible matches were excluded.

HES APC linkage is only available from mid-1997, so it was not possible to ensure that all recorded discharges were cohort members’ first discharges. However, a ‘look back’ period from 1998-2000 was incorporated and people discharged in the first three years of the study period were excluded if an earlier discharge was identified within three years before their index date.

All numbers tabulated are those patients who remain in the cohort after the exclusion criteria have been applied. The SMI cohort are a subset of a broader discharged group used in a previous study, so most exclusions take place prior to the SMI criteria being applied. Patients can have multiple episodes of care within one hospital stay, and/or several episodes across more than one hospital stay. The original data provided by the CPRD contained all episodes for each patient to identify the index episode. Aurum contains records from practices using the EMIS Web® patient record system and GOLD contains records from practices using Vision®.

|  |  | **Aurum Dataset** | | **GOLD Dataset** | |
| --- | --- | --- | --- | --- | --- |
| **Criteria** | **Further detail** | **Patients** | **Patient Episodes** | **Patients** | **Patient Episodes** |
| **HES Cohort delineation** |  |  |  |  |  |
| Records received from CPRD. All episodes of care under a psychiatric consultant between 01/01/1998 and 31/05/2019 | Main Specialty= 710/711/712/713/715 | 485,715 | 1,588,855 | 193,317 | 623,756 |
| **Numbers after exclusions** | | | | | |
| Removal of patients from duplicate practices which have moved electronic record systems (GOLD only) |  | 485,715 | 1,588,855 | 123,461 | 402,426 |
| Keep only ‘ordinary’ inpatients  (Exclude day patients) | (classification!=1) | 484,690 | 1,578,462 | 123,211 | 398,609 |
| Limit to episodes that end with a community discharge | Keep  Discharge destination = 19 Normal place of residence  Or 29 = Temporary place of residence  Or 54,65,66,85 = care homes/foster care  Exclude all transfers and any episodes where the discharge method is 8 (N/A still in hospital) or 4 (died in hospital) | 456,355 | 1,184,355 | 115,812 | 301,716 |
| Limit to discharges within the study period, without recent prior hospitalisation. | Exclude all patients whose  -only discharge falls outside the study period (before 1^st^ January 2001, after 31^st^ May 2018)  -who have a first discharge before the study period and a second within three years of the beginning of the study period. | 365,058 | 799,615 | 92,042 | 202,096 |
| Limit to first discharge | Excludes all subsequent episodes of care to leave one episode per patient | 365,058 | 365,058 | 91,108 | 91,108 |
| Exclude patients with poor quality HES data completion | -episode end and discharge end do not match & either the discharge method or discharge destination is unknown  -exclude those where both the discharge destination and discharge method are unknown.  -drop patients where the episode end date is before the discharge date, or it is missing. | 360,583 |  | 90,979 |  |
| **Linkage Availability** | | | |  |  |
| Exclude patients not eligible for linkage to all necessary data files | -not present in CPRD denominator file  -no linkage with HES,ONS, IMD) | 356,606 |  | 90,150 |  |
| **CPRD Primary Care dataset exclusions** | | | |  |  |
| Exclude patients without acceptable records. | Acceptable!=1 (Aurum)  Accept!=1 (GOLD)  Excludes any with key data missing such as year of birth and gender | 323,526 |  | 84,352 |  |
| Cohort inclusion criteria. | Exclude if:   - Not registered at the GP practice, or had less than 6 months registered at the practice at point of discharge - Age under 10 - Patient recorded at more than one practice at time of discharge (duplicates) | 87,608 |  | 19,795 |  |
| Limit to those with an SMI diagnosis |  |  |  |  |  |
| SMI diagnosis | Keep if primary diagnosis is F20 to F31, F32.3 and F33.3 | 20,717 |  | 4,292 |  |
|  | | | |  |  |
| **Further Exclusions after matching and full linkage to all datasets** | | | |  |  |
| Drop if ONS date of death is before the index date. |  | 20,717 |  | 4,292 |  |
| Drop if case has no matches |  | 20,713 |  | 4,269 |  |
| Drop if case does not meet final quality criteria | -Episode end after discharge date  -Discharge destination is unknown | 20,067 |  | 4,155 |  |
| Excluding those under 18 |  | 19,819 |  | 4,123 |  |
| Final Combined Cohort |  | 23,942 | | | |

**S2. List of diagnostic categories included in the Charlson Comorbidity Index.**

Myocardial infarction

Congestive heart failure

Peripheral vascular disease

Cerebrovascular disease

Dementia

Chronic pulmonary disease

Connective tissue disease

Ulcer disease (peptic)

Mild liver disease

Diabetes without complications

Hemiplegia

Moderate or severe renal disease

Diabetes with complications (end organ damage)

Any tumour, Leukaemia, Lymphoma*

Moderate or severe liver disease

Metastatic solid tumour*

HIV/AIDS

From Khan, N. F., Perera, R., Harper, S., & Rose, P. W. (2010). Adaptation and validation of the Charlson Index for Read/OXMIS coded databases. *BMC Family Practice, 11*(1), 1. Retrieved from https://doi.org/10.1186/1471-2296-11-1.

Original Reference

Charlson, M. E., Pompei, P., Ales, K. L., & MacKenzie, C. R. (1987). A new method of classifying prognostic comorbidity in longitudinal studies: development and validation. *J Chronic Dis, 40*(5), 373-383. doi:10.1016/0021-9681(87)90171-8

**Table S2 - The RECORD statement– checklist of items, extended from the STROBE statement that should be reported in observational studies using routinely collected health data.**

|  | **Item No.** | **STROBE items** | **Location in manuscript where items are reported** | **RECORD items** | **Location in manuscript where items are reported** |
| --- | --- | --- | --- | --- | --- |
| **Title and abstract** | | | | | |
|  | 1 | (a) Indicate the study’s design with a commonly used term in the title or the abstract (b) Provide in the abstract an informative and balanced summary of what was done and what was found |  | RECORD 1.1: The type of data used should be specified in the title or abstract. When possible, the name of the databases used should be included.  RECORD 1.2: If applicable, the geographic region and timeframe within which the study took place should be reported in the title or abstract.  RECORD 1.3: If linkage between databases was conducted for the study, this should be clearly stated in the title or abstract. | Abstract  Geographic region – in abstract  Timeframe – in abstract  Abstract |
| **Introduction** | | | | | |
| Background rationale | 2 | Explain the scientific background and rationale for the investigation being reported |  |  | Introduction: paragraphs 1-4, |
| Objectives | 3 | State specific objectives, including any prespecified hypotheses |  |  | Introduction: paragraph 5 |
| **Methods** | | | | | |
| Study Design | 4 | Present key elements of study design early in the paper |  |  | Methods: ‘Patient population and study design’ |
| Setting | 5 | Describe the setting, locations, and relevant dates, including periods of recruitment, exposure, follow-up, and data collection |  |  | Methods: ‘Data source’ and ‘Patient population and study design’ ‘Appendix p3’ |
| Participants | 6 | *(a) Cohort study* - Give the eligibility criteria, and the sources and methods of selection of participants. Describe methods of follow-up  *Case-control study* - Give the eligibility criteria, and the sources and methods of case ascertainment and control selection. Give the rationale for the choice of cases and controls  *Cross-sectional study* - Give the eligibility criteria, and the sources and methods of selection of participants  *(b) Cohort study* - For matched studies, give matching criteria and number of exposed and unexposed  *Case-control study* - For matched studies, give matching criteria and the number of controls per case |  | RECORD 6.1: The methods of study population selection (such as codes or algorithms used to identify subjects) should be listed in detail. If this is not possible, an explanation should be provided.  RECORD 6.2: Any validation studies of the codes or algorithms used to select the population should be referenced. If validation was conducted for this study and not published elsewhere, detailed methods and results should be provided.  RECORD 6.3: If the study involved linkage of databases, consider use of a flow diagram or other graphical display to demonstrate the data linkage process, including the number of individuals with linked data at each stage. | Methods: ‘Patient population and study design’ ‘Appendix p3’  Codelists available online https://clinicalcodes.rss.mhs.man.ac.uk/  Methods: ‘Patient population and study design’ ‘Appendix p3’ |
| Variables | 7 | Clearly define all outcomes, exposures, predictors, potential confounders, and effect modifiers. Give diagnostic criteria, if applicable. |  | RECORD 7.1: A complete list of codes and algorithms used to classify exposures, outcomes, confounders, and effect modifiers should be provided. If these cannot be reported, an explanation should be provided. | Methods: ‘Classification of outcomes and covariates’  Codelists available online https://clinicalcodes.rss.mhs.man.ac.uk/ |
| Data sources/ measurement | 8 | For each variable of interest, give sources of data and details of methods of assessment (measurement).  Describe comparability of assessment methods if there is more than one group |  |  | Methods: ‘data sources’ and ‘Classification of outcomes and covariates’ ‘Appendix p2’ |
| Bias | 9 | Describe any efforts to address potential sources of bias |  |  | Methods: ‘Patient population and study design’ ‘Appendix p3’ |
| Study size | 10 | Explain how the study size was arrived at |  |  | Methods: Patient population and study design’ ‘Appendix p3’ |
| Quantitative variables | 11 | Explain how quantitative variables were handled in the analyses. If applicable, describe which groupings were chosen, and why |  |  | Methods: Classification of outcomes and covariates’ and ‘statistical analysis’ |
| Statistical methods | 12 | (a) Describe all statistical methods, including those used to control for confounding  (b) Describe any methods used to examine subgroups and interactions  (c) Explain how missing data were addressed  (d) *Cohort study* - If applicable, explain how loss to follow-up was addressed  *Case-control study* - If applicable, explain how matching of cases and controls was addressed  *Cross-sectional study* - If applicable, describe analytical methods taking account of sampling strategy  (e) Describe any sensitivity analyses |  |  | Methods: Statistical analyses (a,b)  Classification of outcomes and covariates (c), appendix p3  Patient population and study design’ (c,d) |
| Data access and cleaning methods |  | .. |  | RECORD 12.1: Authors should describe the extent to which the investigators had access to the database population used to create the study population.  RECORD 12.2: Authors should provide information on the data cleaning methods used in the study. | Methods: ‘Patient population and study design’ and ‘Role of funding source’  Appendix p3 |
| Linkage |  | .. |  | RECORD 12.3: State whether the study included person-level, institutional-level, or other data linkage across two or more databases. The methods of linkage and methods of linkage quality evaluation should be provided. | Methods: ‘Data source’ |
| **Results** | | | | | |
| Participants | 13 | (a) Report the numbers of individuals at each stage of the study (*e.g.*, numbers potentially eligible, examined for eligibility, confirmed eligible, included in the study, completing follow-up, and analysed)  (b) Give reasons for non-participation at each stage.  (c) Consider use of a flow diagram |  | RECORD 13.1: Describe in detail the selection of the persons included in the study (*i.e.,* study population selection) including filtering based on data quality, data availability and linkage. The selection of included persons can be described in the text and/or by means of the study flow diagram. | Methods; ‘Patient population and study design’, appendix p3 and results paragraph 1. |
| Descriptive data | 14 | (a) Give characteristics of study participants (*e.g.*, demographic, clinical, social) and information on exposures and potential confounders  (b) Indicate the number of participants with missing data for each variable of interest  (c) *Cohort study* - summarise follow-up time (*e.g.*, average and total amount) |  |  | Results ‘Descriptive information’ , Table 1, Appendix Table S3 (c) |
| Outcome data | 15 | *Cohort study* - Report numbers of outcome events or summary measures over time  *Case-control study* - Report numbers in each exposure category, or summary measures of exposure  *Cross-sectional study* - Report numbers of outcome events or summary measures |  |  | Results, Table 2. |
| Main results | 16 | (a) Give unadjusted estimates and, if applicable, confounder-adjusted estimates and their precision (e.g., 95% confidence interval). Make clear which confounders were adjusted for and why they were included  (b) Report category boundaries when continuous variables were categorized  (c) If relevant, consider translating estimates of relative risk into absolute risk for a meaningful time period |  |  | Results, figure 2,3 appendix p 12 |
| Other analyses | 17 | Report other analyses done—e.g., analyses of subgroups and interactions, and sensitivity analyses |  |  | Results, paragraph 2 and 4. Table 3, figure 3. |
| **Discussion** | | | | | |
| Key results | 18 | Summarise key results with reference to study objectives |  |  | Discussion, paragraph 1 |
| Limitations | 19 | Discuss limitations of the study, taking into account sources of potential bias or imprecision. Discuss both direction and magnitude of any potential bias |  | RECORD 19.1: Discuss the implications of using data that were not created or collected to answer the specific research question(s). Include discussion of misclassification bias, unmeasured confounding, missing data, and changing eligibility over time, as they pertain to the study being reported. | Discussion, paragraph 7 |
| Interpretation | 20 | Give a cautious overall interpretation of results considering objectives, limitations, multiplicity of analyses, results from similar studies, and other relevant evidence |  |  | Discussion: paragraphs 2-5, |
| Generalisability | 21 | Discuss the generalisability (external validity) of the study results |  |  | Discussion paragraph 8 |
| **Other Information** | | | | | |
| Funding | 22 | Give the source of funding and the role of the funders for the present study and, if applicable, for the original study on which the present article is based |  |  | Methods, ‘Role of funding source’, ‘Acknowledgements’ |
| Accessibility of protocol, raw data, and programming code |  | .. |  | RECORD 22.1: Authors should provide information on how to access any supplemental information such as the study protocol, raw data, or programming code. | ‘Availability of data and materials’ |

*Checklist is protected under Creative Commons Attribution ([CC BY](http://creativecommons.org/licenses/by/4.0/)) license.

Reference: Benchimol EI, Smeeth L, Guttmann A, et al. The REporting of studies Conducted using Observational Routinely-collected health Data (RECORD) Statement. *PLOS Medicine* 2015; **12**(10): e1001885.

**Figure S1 - Age distribution of SMI subgroup**

**Figure S2 Cumulative incidence percentage values (and their 95% confidence intervals) of all-cause mortality in the first year post-discharge compared to individuals with a diagnosis of an SMI but without recent hospital admission.** *Light grey, discharged SMI cohort, dark grey, community SMI comparison cohort.*


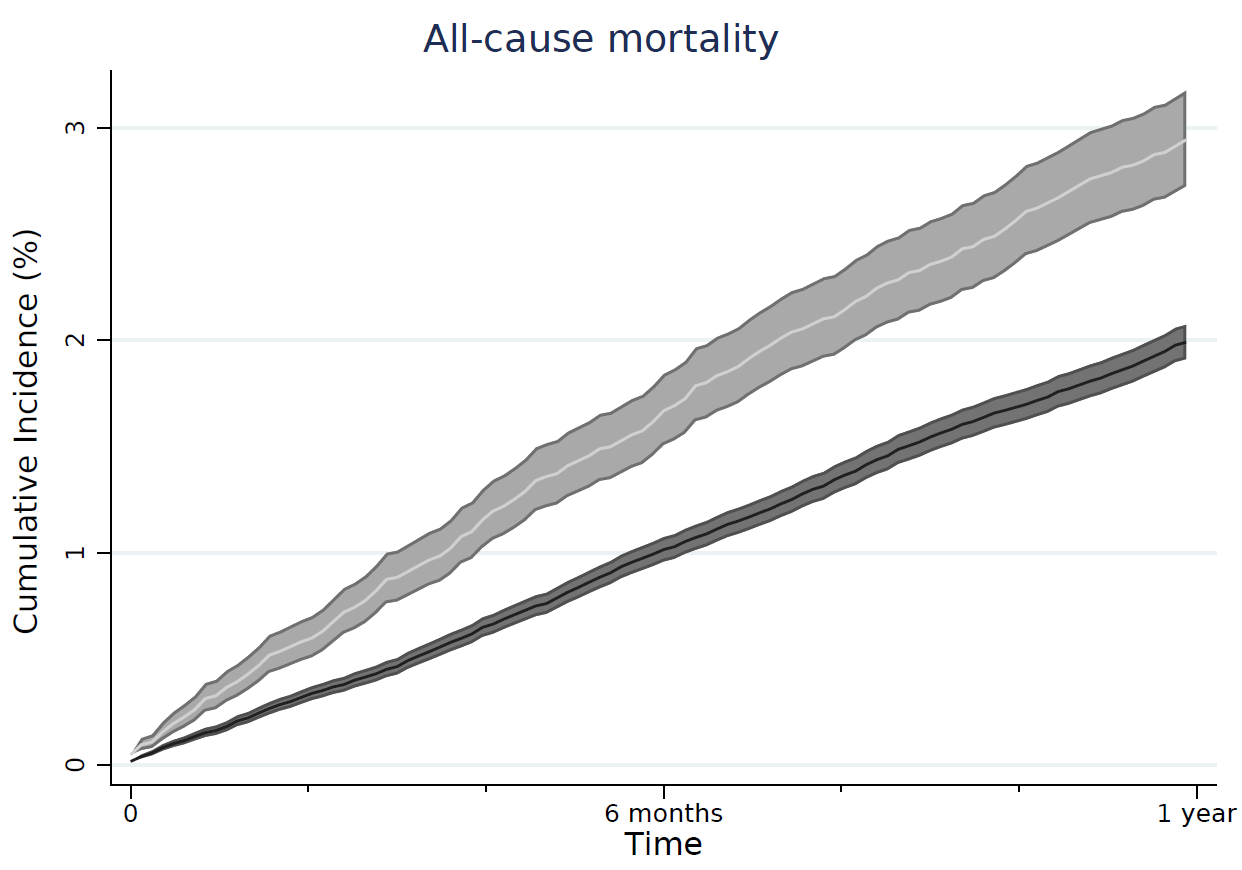


**Figure S3 Cumulative incidence percentage values (and their 95% confidence intervals) of natural causes of death in the first year post-discharge compared to individuals with a diagnosis of an SMI but without recent hospital admission.** *Light grey, discharged SMI cohort, dark grey, community SMI comparison cohort.*
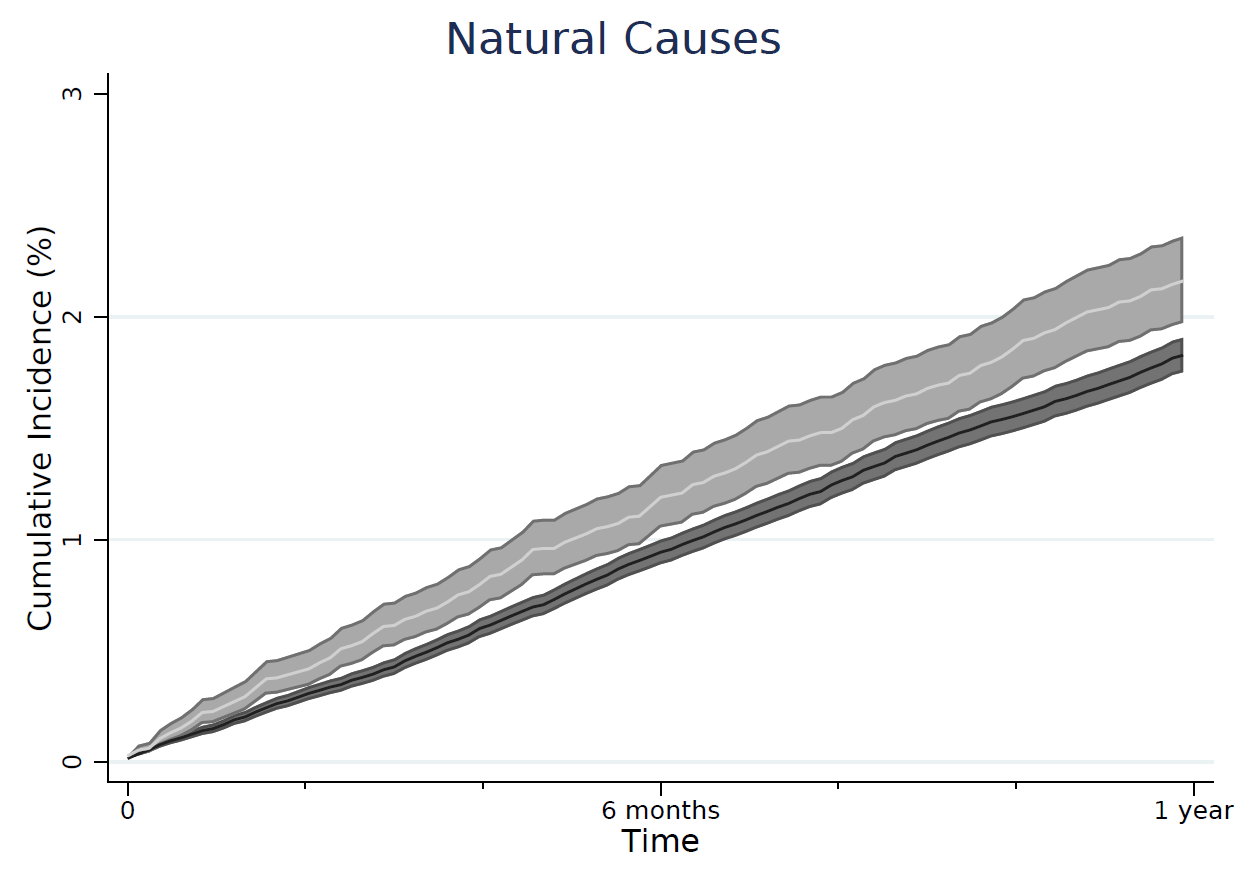


**Figure S3 Cumulative incidence percentage values (and their 95% confidence intervals) of external causes of death in the first year post-discharge compared to individuals with a diagnosis of an SMI but without recent hospital admission.** *Light grey, discharged SMI cohort, dark grey, community SMI comparison cohort.*


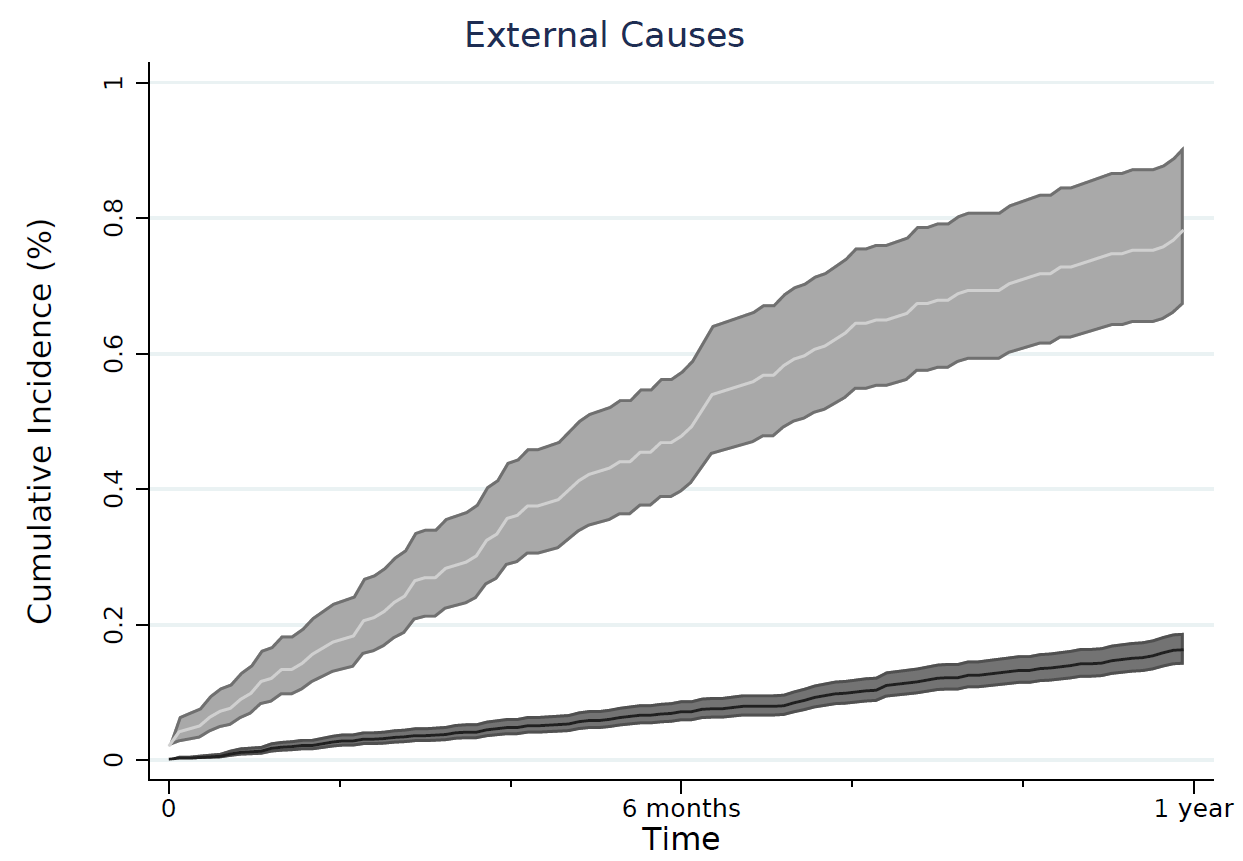


**Figure S2 – Graph showing the cumulative incidence of suicide in the first year after discharge.** Line shows suicides by all individuals discharged from the care of a psychiatric consultant irrespective of diagnosis with confidence intervals (n=100,761).


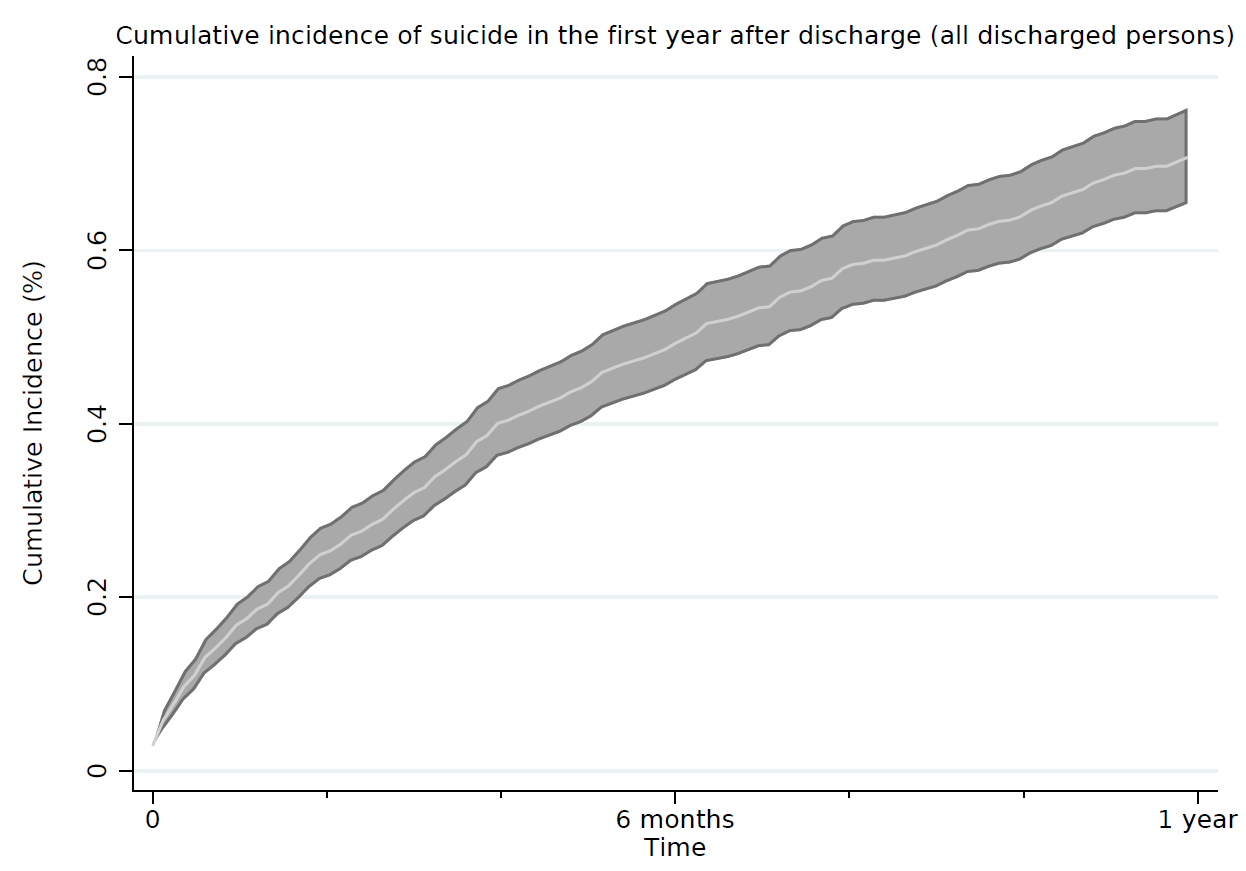


**Table S3 – Unadjusted and adjusted hazard ratios by cause of death and follow-up time**

|  |  |  |  |  | Unadjusted | | | Model 1 *(adjusted for IMD, SMI subgroup)* | | | Model 2 a *(adjusted for IMD, SMI subgroup, previous self-harm)* | | Model 2 b *(adjusted for IMD, SMI subgroup, comorbidity)* | | Model 3 *(adjusted for IMD, SMI group, previous self-harm, comorbidity)* | |
| --- | --- | --- | --- | --- | --- | --- | --- | --- | --- | --- | --- | --- | --- | --- | --- | --- |
| Cause of death | Follow up time | Person years at risk | Deaths (Primary Cohort) | Deaths (Community cohort) | Hazard Ratio (95% CI) | | | Hazard Ratio  (95% CI) | | | Hazard Ratio  (95% CI) | | Hazard Ratio  (95% CI) | | Hazard Ratio  (95% CI) | |
| Suicide |  |  |  |  |  | |  |  | |  |  |  |  |  |  |  |
|  | 0-3mths | 35,108 | 45 | 24 | 9.7 | (5.8-16) | | 11.6 | (6.4-20.9) | | 10.4 | (5.7-19) |  |  | 10.4 | (5.7-19) |
|  | 3-12mths | 99,715 | 83 | 73 | 6.2 | (4.5-8.6) | | 6.5 | (4.6-9.2) | | 6.0 | (4.2-8.5) |  |  | 5.9 | (4.1-8.4) |
|  | 1-2 years | 119,869 | 47 | 75 | 3.4 | (2.3-5.1) | | 3.7 | (2.4-5.6) | | 3.3 | (2.2-5.1) |  |  | 3.3 | (2.2-5.1) |
|  | 2-5 years | 278,932 | 72 | 190 | 2.2 | (1.7-3) | | 2.3 | (1.7-3.2) | | 2.1 | (1.5-2.8) |  |  | 2.1 | (1.5-2.8) |
| Natural causes | |  |  |  |  |  | |  |  | |  |  |  |  |  |  |
|  | 0-3mths | 35,108 | 138 | 491 | 1.5 | (1.3-1.9) | | 1.6 | (1.3-1.9) | |  |  | 1.7 | (1.4-2.1) |  |  |
|  | 3-12mths | 99,715 | 325 | 1,613 | 1.2 | (1-1.3) | | 1.2 | (1-1.3) | |  |  | 1.2 | (1.1-1.4) |  |  |
|  | 1-2 years | 119,869 | 312 | 2,006 | 0.9 | (0.8-1) | | 0.9 | (0.8-1.1) | |  |  | 1.0 | (0.9-1.1) |  |  |
|  | 2-5 years | 278,932 | 882 | 4,440 | 1.2 | (1.1-1.3) | | 1.2 | (1.1-1.3) | |  |  | 1.3 | (1.2-1.4) |  |  |
| All-cause mortality | |  |  |  |  |  | |  |  | |  |  |  |  |  |  |
|  | 0-3mths | 35,108 | 198 | 534 | 2.0 | (1.7-2.4) | | 2.1 | (1.8-2.5) | | 2.1 | (1.8-2.5) | 2.2 | (1.9-2.7) | 2.3 | (1.9-2.7) |
|  | 3-12mths | 99,715 | 434 | 1,757 | 1.4 | (1.3-1.6) | | 1.5 | (1.3-1.6) | | 1.4 | (1.3-1.6) | 1.5 | (1.4-1.7) | 1.5 | (1.3-1.7) |
|  | 1-2 years | 119,869 | 380 | 2,178 | 1.0 | (0.9-1.1) | | 1.1 | (0.9-1.2) | | 1.0 | (0.9-1.2) | 1.1 | (1-1.2) | 1.1 | (1-1.2) |
|  | 2-5 years | 278,932 | 1,004 | 4,843 | 1.3 | (1.2-1.4) | | 1.3 | (1.2-1.4) | | 1.3 | (1.2-1.4) | 1.3 | (1.2-1.4) | 1.3 | (1.2-1.4) |
| External causes | |  |  |  |  |  | |  |  | |  |  |  |  |  |  |
|  | 0-3mths | 35,108 | 60 | 43 | 7.2 | (4.8-10.7) | | 8.0 | (5.2-12.4) | | 7.5 | (4.8-11.7) |  |  | 7.5 | (4.8-11.7) |
|  | 3-12mths | 99,715 | 109 | 144 | 4.2 | (3.3-5.5) | | 4.2 | (3.2-5.5) | | 3.8 | (2.9-5) |  |  | 3.8 | (2.9-5) |
|  | 1-2 years | 119,869 | 68 | 172 | 2.2 | (1.6-3) | | 2.2 | (1.7-3) | | 2.0 | (1.5-2.8) |  |  | 2.1 | (1.5-2.9) |
|  | 2-5 years | 278,932 | 122 | 403 | 1.9 | (1.5-2.3) | | 1.9 | (1.5-2.4) | | 1.7 | (1.3-2.2) |  |  | 1.7 | (1.4-2.2) |
